# Activities supporting the growth of Clinical Trial Networks in Australia

**DOI:** 10.1186/s13063-021-05974-3

**Published:** 2022-01-28

**Authors:** Fiona Nemeh, Rachelle Buchbinder, Carmel M. Hawley, Mark R. Nelson, Jacqui G. Waterkeyn, Christopher M. Reid

**Affiliations:** 1 Australian Clinical Trials Alliance, Melbourne, Victoria Australia; 2 Department of Epidemiology and Preventive Medicine, School of Public Health and Preventive Medicine, Monash University, Prahran, Victoria Australia; 3 Monash Department of Clinical Epidemiology, Cabrini Institute, Malvern, Victoria Australia; 4 Department of Nephrology, Princess Alexandra Hospital, Brisbane, Australia; 5 Australasian Kidney Trials Network, Faculty of Medicine, University of Queensland, Brisbane, Australia; 6 Translational Research Institute, Brisbane, Australia; 7 School of Public Health and Preventive Medicine, Monash University, Prahran, Victoria Australia; 8 Menzies Institute for Medical Research, University of Tasmania, Hobart, Tasmania Australia; 9 Orygen, Parkville, Victoria Australia; 10 School of Public Health, Curtin University, Bentley, Western Australia Australia

**Keywords:** Collaboration, Clinical trial, Clinical Trial Network, Establish, Roadmap

## Abstract

Clinical Trial Networks in which trialists work collaboratively enable multi-site, large-scale, high-quality clinical trials to be efficiently run. Although the benefits of Clinical Trial Networks are largely known, establishing a Clinical Trial Network can be complex. There are many factors for clinicians and researchers to consider, and there is currently a paucity of information on how to form a Clinical Trial Network. This article provides a suggested roadmap on how to establish a Clinical Trial Network. The Australian Clinical Trials Alliance (ACTA) is the peak body for Clinical Trial Networks, Coordinating Centres and Registries in Australia, and has produced several resources to support the effective and efficient running of clinical trials. This guide has come about through discussions with members of the ACTA Clinical Trial Network Sector Expansion Reference Group consisting of clinical trialists, clinicians, researchers, and consumers.

## Background

A Clinical Trial Network (CTN) is a group of clinicians, health professionals, researchers, and often consumers who collaborate to conduct clinical trials in a defined disease or discipline. CTNs conduct and publish multi-site clinical trials, to strengthen and improve the evidence base for high-quality health care and/or increase the uptake of evidence into practice. Trials conducted by CTNs are more likely to influence clinical guidelines and government directives due to their rigour, size, and power [1]. They may also be more likely to identify the most important or pertinent research questions, and they encourage the efficient use of resources [2, 3]. For these reasons, CTNs tend to have higher rates of success with grants, and findings are often published in high profile medical journals [4–7].

According to ACTA’s guidance document on activities critical to success [8], CTNs are exemplars of the integration between research and healthcare delivery and can utilise capabilities to maximise the delivery of trial outputs to benefit patients. CTNs provide greater capacity for generation of evidence to enhance external validity of trial outcomes. CTNs provide the opportunity to build a ‘brand’ that has a reputation for delivering trials on time and within budget, high-impact trials that answer questions of importance to clinicians and patients and the ability to attract and maintain research funding [8].

The core principles of most CTNs include [adapted from ACTA’s CTN establishment guide ([9], p. 6):

• Collaboration and collegiality including sharing credit for success (e.g. group authorship and mutual ownership of outcomes and achievements)

• Alignment of the best interests of researchers, patients, and the CTN

• Equity

• Creation of a reusable, sustainable, shared infrastructure that improves trial quality and feasibility

• Commitment to improving patient outcomes through generating and implementing evidence derived from trials

• Conduct of high-quality clinical trials that are patient-centred and innovative

• Enhancing efficiency of research through coordination of potentially competing trials and prioritisation of research questions

In Australia, there are now over 40 CTNs in a range of areas including intensive care, anaesthesia [7], and musculoskeletal [10]. The CTNs are at various stages of development and many of the more established and successful CTNs have published numerous landmark clinical trials over the last decade [4, 11–14]. A key example is the ATACAS (Aspirin and Tranexamic Acid for Coronary Artery Surgery) trial [15–17]. In this landmark study, over 4600 participants were enrolled across Australia, New Zealand, Asia, and Europe. Findings from the ATACAS study will guide the routine clinical care of patients undergoing coronary artery surgery [15].

## CTNs and COVID-19

Importantly, in the current health climate of 2021, the existence of established CTNs in areas such as intensive care and anaesthesia have provided the infrastructure to enable rapid testing of coronavirus disease 19 (COVID-19) treatments on a large scale. Examples of clinical trials conducted by CTNs in Australia for COVID-19 are the Randomised Embedded Multifactorial Adaptive Platform for Community-Acquired Pneumonia (REMAP-CAP) study [18] led by Professor Steven Webb from the Australian and New Zealand Intensive Care Society Clinical Trials Group (ANZICS-CTG) and the Australasian COVID-19 Trial (ASCOT) [19] led by Associate Professor Steven Tong from the Australasian Society for Infectious Diseases Clinical Research Network (ASID-CRN).

REMAP-CAP [18] is examining different antibiotics, duration of macrolide therapy, glucocorticoid treatment, and different approaches to mechanical ventilation in patients with COVID-19. The ASCOT trial is investigating if using antiviral therapies and therapeutic antibody treatment can prevent the need for ventilation in patients with COVID-19 [19]. The REMAP-CAP and ASCOT studies show that CTNs have played a key role in enabling clinical trials to be adapted to address the pandemic and collaboration with international trialists has helped to advance this work due to the low incidence of COVID-19 in Australia.

To date, there has been limited information for those who wish to form a CTN [10, 20, 21]. This article presents a suggested roadmap on how to form and progress the establishment of a CTN. This roadmap includes considerations of benefits and barriers to forming a CTN, governance, network operational models, infrastructure and human resources, business structure, and finance.

## Benefits and barriers to forming a CTN

### Benefits

The potential benefits of a CTN need to be demonstrated early to stakeholders [22]. One benefit might be to increase the ability to attract funding [23] or to undertake more influential studies [20]. Established CTNs often interact and collaborate on clinical trials with international network groups [24]. Another drawcard might be the clinical relevance of a research question and its importance to members [10]. Often these questions can only be answered with a sufficient sample size and power to address clinical outcomes through a collaborative CTN [25]. As an emerging researcher/clinician, participating in network-based trials may provide the potential for career progression [20]

CTNs contribute substantially to making the health care system more efficient and effective as collaboration brings new treatments to more patients faster. Features that contribute to this are shown in Table 1 below (adapted from sector gap analysis [26]; p. 5).

**Table 1:** Features of CTNs which contribute to an efficient and effective health care system

• Creating a national community of practice that is comprised of clinicians, clinical researchers, and consumers who have a shared mission to improve patient care through clinical trials. • Identification of research questions that are most relevant to practice and policy because clinician-researchers and consumers are also end-users of evidence generated by their trials. • Enabling peer- and consumer-review, often within formal endorsement processes, to develop high-quality (valid, feasible, and relevant) clinical trial proposals. • Access to a geographically diverse, large, and representative patient population to facilitate recruitment to well-powered and harmonised clinical trials. • Sharing experience in the design and conduct of cost-effective clinical trials. • Providing capacity for enhanced translation and implementation of clinical trial findings. • Establishing efficient and reusable infrastructure to support multiple clinical trials and embedding learnings moving forward. • Providing a supportive environment for the training and employment of future researchers including teaching cutting edge new methods and how to do systematic reviews to inform the best research questions. • Providing operational support for members doing trials including compliance, site visits, and audits.	

According to ACTA’s Establishment of New Clinical Trial Networks Guidance document [9], there are many benefits of a CTN that include but are not limited to:

• Quality, efficiency, and impact of trials are strengthened

• Minimum endorsement criteria are established

• Skills, processes, and systems of the CTN can be drawn on by members

• A greater sample size can be accessed through the collaboration of multiple trial sites with the capacity of rapid expansion of evidence base, improved recruitment of trial participants and larger sample sizes that enable smaller differences in interventions to be detected

• Peer review of projects for feasibility and validity and manuscripts prior to submission to journals

• Standardised trial outcome measures are developed and agreed

• A consumer advisory panel for consumers to give input on the suitability of candidate interventions for trials, research priorities, outcome measures, and trial protocols

• Increased data integrity gained through good clinical practice implementation

• Through consultation of a CTNs broadly representative membership, CTNs can foster access to and sharing of novel research methods, advances in the disease area, trial design, and strategies that benefit the therapeutic area of the CTN

• Multiple trials can be run and managed at once

• Clinician-led design and prioritisation of trials that can be embedded into healthcare by drawing on existing treatment protocols, response measurements, and resources, which ultimately enhances translation of trial results into routine healthcare

• Development of infrastructure that can be reused for subsequent trials including robust site systems, site feasibility assessment and selection, training in research data capture, Case Report Form development and standardisation, electronic Data Capture (eDC) systems, budget negotiation, and project management including Clinical Trial Management

• Systems, sponsor-delegated monitoring, and other clinical trial capabilities that are not always readily available at individual institutions

• CTNs can provide operational procedures to maintain regulatory compliance, independent Project Managers and monitors for studies

• Development of standardised study tools

• Maximising research capacity by inclusion of additional complementary studies to enhance research into the disease area

Although many trials, including some high-impact trials, are conducted without a CTN, networks enable sharing of experience, processes, infrastructure, and tools in a more structured manner so that ‘reinventing the wheel’ is avoided. This along with factors identified creates a synergy where a network is greater than the sum of its parts, enabling efficiencies and effectiveness that would not be possible without a CTN [8].

### Barriers

Initially people may have concerns about establishing a network. There may be perceived threats to independence and fear of exploitation or taking over of locally established initiatives and smaller groups [22]. Protection of these local resources is often seen as a barrier to a national or collaborative approach to establishing networks. Researchers may also be concerned about having their ideas taken by others. A clear Vision, Mission and Values statement and open and transparent communication can address these issues [10]. Other possible disadvantages of CTNs that may be barriers are a risk to the reputation of all members if a trial fails or by actions of other members [9]. Resources to run central activities need to be sourced and may divert resources from individuals or groups [9]. The next sections will provide guidance on forming or progressing a CTN.

## A ‘recipe’ for setting up or improving an existing CTN

### Stakeholders

The type of stakeholders needed for a CTN depends on what kind of model the network adopts. There is a need to consider internal versus external stakeholders. Who will be part of the group and what roles will be outsourced? For some positions, funding is required; others might be in kind. For further information, see ACTA’s stakeholder mapping tool [22]. After stakeholders have been engaged, the governance structure of a CTN should be considered.

### CTN governance

Emerging CTNs can be unsure about the best way to set up their governance structure. The structure of the committees will depend on the model of the network. Deciding on a Vision, Mission and Values statement then creating a Terms of Reference (ToR) can help define the role of different committees such as scientific subcommittees and governance bodies. Consider which committees are needed for the set-up and operationalising of the network such as a consumer advisory group, a scientific advisory group, and a CTN executive. Later additions to a CTN might include a common safety review board or joint data safety management board, an external advisory board, subspeciality interest groups, and an early career fellow group. Consider committee needs such as size, membership, knowledge base, and operational expertise and what will constitute a quorum. See ACTA’s guidance on governance structures for further information [27]**.**

### Network operational models

Different models of CTN activity include Facilitating and Coordinating roles. *Facilitating CTNs* have little or no direct role in the day to day running of clinical trials but provide opportunity for collaborative development, central negotiating of trial budgets, and funding allocation [9]. Instead, trials are run through specialist trial coordinating centres which are based in universities or medical research institutions. Governance is independent of the CTN and Facilitating CTNs do not act as the study sponsor. A *Coordinating CTN* represents the evolution and growth of a facilitating CTN, i.e. Coordinating CTNs actively conduct trials, have broader Good Clinical Practice (GCP) responsibilities, and provide direct project management for trials, site management, recruitment mediation, monitoring, data management, statistical analysis, and regulatory compliance [9]. The institution that hosts the coordinating CTN can act as the trial sponsor or the CTN itself can sponsor trials [9]. Both facilitating and coordinating models (or a hybrid of both) appear to be effective [28]. See Table 2 for the differences between facilitating and coordinating CTNs and Table 3 for corporate considerations for facilitating or coordinating CTNs.

**Table 2:** Activities for facilitating and coordinating CTNs ([29], p. 13)

Potential activities for clinical trial facilitation	Additional activities for clinical trial coordination
Identification of important clinical questions	Direct trial coordination and management by CTN
Collaborative study protocol development	Site management
Peer review and formal endorsement of trials	Data management
Scientific meetings	Recruitment of trial participants
Grant writing	Monitoring
Education/training/mentoring of researchers	Statistical analysis
Advocacy and industry/consumer liaison	Regulatory compliance
Site selection and trial oversight	May or may not act as study Sponsor
Clinical guideline development

**Table 3:** Corporate considerations for facilitating or coordinating CTNs

If an answer to any of below is ‘no’, CTN may be better suited to facilitating model	Yes or No
1. Does the CTN want to take on role of GCP Sponsor and/or offer a trial coordinating centre service (internal/outsourced) to its members?	
2. Can the CTN access funding/develop sufficient trials to establish and maintain a trial centre? -Need at least 2 people or 1.2 FTE to cover leave etc. even if only one trial. -n/a if outsourcing trial conduct.	
3. Does the CTN have industry-experienced personnel that will allow simultaneous development of CTN-specific processes while not delaying trial start up?	
4. Is expertise and resource available in the CTN for some or all of the following: statistical planning and analysis, database programming, data management, trial coordination, and (possibly) abiding by GCP Sponsor requirements? Providing secure data management can be expensive.	
5. Can the CTN provide clinical trial insurance (this may also be provided by the trial sponsor for IITs as well as local Institutional insurance)?	
6. Will the CTN need to be a legal entity if acting as sponsor?	

*CTN* Clinical Trial Network, *FTE* full-time equivalent, *GCP* Good Clinical Practice, *IIT* investigator-initiated trial

### Infrastructure and human resources for a CTN

Clinical trial infrastructure can be slow to bring up to the level needed to initiate a site. Similarly, not all institutions and smaller commercial organisations have the skills, expertise, or resources to sponsor trials. These organisations often need to find funding to obtain this knowledge and experience, either through their own resources or contracting a Contract Research Organisation (CRO). Centrally providing some of this capacity through a CTN can save money, expedite start up, and ensure the compliance framework for running trials are met.

A CTN must have basic infrastructure and human resources applicable to the stage of development. Even in successful CTNs, there needs to be a lot of negotiation for resources, quality systems, bioinformatics, and staff such as project managers and monitors. However, CTNs may initially only have an Executive Officer. Some groups do not fund their own trials but provide expertise and endorsement for others, depending on the CTN model and funding opportunities. Sometimes registries are embedded in a network which adds another layer of complexity to network models. There can be several decisions to be made when setting up as CTN. Will a network be a Facilitating or Coordinating CTN? Is there a need for a central infrastructure base and how many full-time equivalent personnel are required from CTN establishment through stages of development year 1 to *x*.

### Business structure of a CTN

**Fig. 1:**
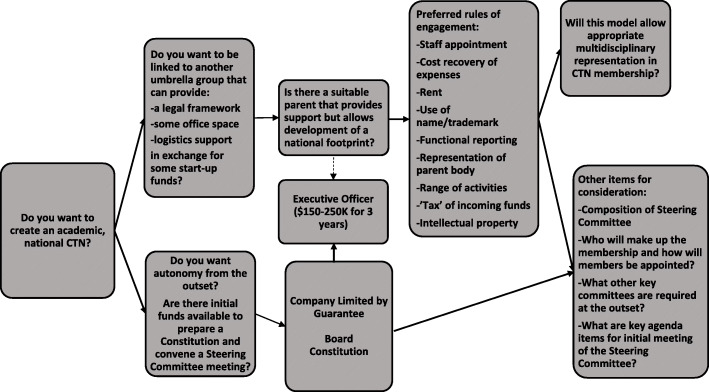
Considerations for the business structure of a CTN

There is also a need to consider the business structure of a network, if and when it will become an independent corporate entity. Some CTNs are part of clinical societies so are owned by them—others are multidisciplinary. There are pros and cons of being owned. For example, if a CTN is multidisciplinary no one owns the network, but this can make it hard for sign off on CTN matters. Figure 1 shows considerations for the operational business structure of a CTN. However, it is important to seek financial advice as part of this process.

### Financing a CTN

Emerging CTN groups often want to know how to obtain startup funding and develop a sustainable funding model for a CTN. Professional and consumer organisations will sometimes provide startup funding. For example, the Australia and New Zealand Musculoskeletal Clinical Trials Network (ANZMUSC) approached some professional societies and consumer organisations to provide funds for their first meeting [10]. Some other possible sources of local funding for networks might be through a National Health and Medical Research Council (NHMRC) funding opportunity, such as a Centre for Research Excellence (CRE), Medical Research Future Fund (MRFF), or an Australian Research Council (ARC)-linkage grant. A CRE grant enabled establishment of the paediatric emergency department research, musculoskeletal, and cardiovascular CTNs [10]. However, in Australia, grants for clinical trials are usually only 3 to 5 years in duration. This recurrent competitive funding model often results in the loss of staff and knowledge at the end of a grant cycle.

### Other clinical trial funding models

In the United Kingdom (UK), funding for research and clinical trials is integrated into the health care system through the National Institute of Health Research (NIHR) [30, 31]. The ongoing financial support from the government has provided opportunities for patients to join clinical trials and for general practices to undertake research projects. The UK model highlights the success of implementing funding for clinical trials into the healthcare system. Fifteen local Clinical Research Networks (CRNs) have been established and deliver research across 31 specialties [32]. In 2019/2020, 150,000 participants were recruited into studies in primary care and one third of all English general practices recruited participants [32]. Ongoing financial support for clinical trials in the UK has resulted in increased enrolment, reduced costs, and faster completion of trials thereby improving delivery of research. It has been argued that this UK model for clinical trials should be implemented in Australia [1] or a percentage of total healthcare be allocated to funding clinical trials [33].

### Overseas funding

Overseas funding opportunities should also be considered. Recently, the Wellcome Trust, a philanthropic organisation in the UK, provided funding for the establishment of the Australian Early Psychosis Collaborative Consortium (AEPCC) Clinical Registry and Clinical Trial and Translation Network (CTTN) [34]. This grant will support research into youth mental health, particularly the onset and treatment of psychosis in young people. The National Institutes of Health (NIH) in the United States (US) also provides overseas funding opportunities for health researchers. The ASPirin in Reducing Events in the Elderly (ASPREE) and the ASPREE extension study (ASPREE-XT) obtained funding through the NIH [35].

### Industry funding

A further option may be to engage with industry to support the network. For example, industry may come to a CTN to access network recruitment. This supplemental funding can help make a CTN sustainable in the longer term. The Australasian Stroke Trials Network (ASTN) and Australian Epilepsy Clinical Trials Network (AECTN) have both leveraged industry trials for sustainability [28]. The structure of funding is constantly changing and needs to change with the life of the CTN. It is suggested that emerging CTNs keep watch for funding opportunities and be prepared to respond at short notice.

Funding can depend on the CTN area and membership base at different stages of development (see Table 4 for stages of establishment (Australian Clinical Trials Alliance (ACTA): CTN establishment progress, Unpublished)). Table 4 provides a suggested framework for leaders within a discipline to advance through the stages that lead to establishment of an effective and sustainable CTN. With the establishment of ACTA, they are able to facilitate this process. Typically, milestones of CTN establishment include the following, but note that this is only one method and some of the steps may occur concurrently:

**Table 4:** Suggested activities to facilitate CTN establishment (Australian Clinical Trials Alliance (ACTA): CTN establishment progress, Unpublished)

Stage of establishment	Activity		Milestone
**Facilitation**	Discussions are held between a group of leaders in the field who share an interest in developing a CTN. Typically held by videoconference.	1.	Agreement that establishment of a CTN should be proposed to wider audience and why a CTN would be beneficial prior to further stakeholder consultations.
	Meeting with wide group of stakeholders and interested parties, often held face to face.	2.	Agreement that establishment of a CTN should progress and discussion whether it should be facilitating or coordinating.
	Assessment of how CTN will operate in existing sector.	3.	Strategy for engagement of colleges, societies, overlapping CTNs, registries, industry, philanthropic organisations, and advocacy groups. May include preliminary discussion of options regarding location of central CTN operations and seed funding.
**CTN structure**	Governance structure discussed including representation of sub-speciality areas and other relevant disciplines.	4.	Finalisation of initial governance/steering committee and written Terms of Reference.
	Consideration of the options for membership structures.	6.	Formalisation of membership structure and strategy for engagement of health service providers and other important stakeholders. System for recording members established.
	Business structure discussed and agreed.	7.	Business structure formalised with appropriate documentation (Agreement with parent organisation, constitution, etc.).
	Assessment of existing clinical trial infrastructure.	8.	Decision whether to proceed as a facilitating or coordinating CTN.
	Meeting of governance committee for strategic planning and discussion of mission, vision, and values.	9.	Formalisation of strategic plan.
**CTN launch**	Commencement of administration activities.	10.	Seed funding sourced and appointment of an Executive Officer^a^.
	Communications strategy.	12.	Launch of website/distribution of first member newsletter.
	Criteria for endorsement of trials by CTN and authorship criteria discussed and agreed.	13.	Formalisation of authorship and endorsement policy with or without prioritisation.
**Clinical trial activities**	Trial proposals considered.	14.	Endorsement of first trial.
	Commencement of clinical trial conduct.	15.	Conduct foundation trial.
	Meeting of the membership.	16.	Inaugural annual scientific meeting.
**Wider membership engagement**	Strategies to engage the wider membership. May include meetings of sub-diseases or disciplinary areas, special interest areas, educational workshops, for formal mentoring, etc.	17.	Smaller meeting or mentoring programme established.

^a^Appointment of an Executive Officer greatly facilitates CTN establishment but not all CTNs will be able to access sufficient seed funding to achieve this milestone. Despite not appointing an Executive Officer, the CTN may continue to achieve other milestones.

## Conclusions

By working collaboratively and sharing existing infrastructure, CTNs enable the ability to attract funding and undertake more influential studies. This can lead to the generation of better evidence that can be embedded in the healthcare system. Benefits of CTNs for members and patients are well documented. However, barriers to forming a CTN such as cost and perceived threats to independence can increase the difficulty of this process. It is hoped that by providing a roadmap for clinical trialists considering setting up a CTN, the process will be easier as there are resources to guide this journey.

## Data Availability

Not applicable.

## Abbreviations

ACTA: Australian Clinical Trials Alliance
AECTN: Australian Epilepsy Clinical Trials Network
ANZICS-CTG: Australian and New Zealand Intensive care Society Clinical Trials Group
ANZMUSC: Australia and New Zealand Musculoskeletal Clinical Trials Network
ARC: Australian Research Council
ASCOT: Australasian COVID-19 Trial
ASID-CRN: Australasian Society for Infectious Diseases Clinical Research Network
ASPREE: ASPirin in Reducing Events in the Elderly
ASPREE-XT: ASPREE Extension Study
ASTN: Australasian Stroke Trials Network
ATACAS: Aspirin and Tranexamic Acid for Coronary Artery Surgery
COVID-19: Coronavirus disease 2019
CRE: Centre for Research Excellence
CRN: Clinical Research Network
CRO: Contract Research Organisation
CTN: Clinical Trial Network
CTTN: Clinical Trial and Translation Network
eDC: Electronic Data Capture
GCP: Good Clinical Practice
IIT: Investigator-initiated trial
MRFF: Medical Research Future Fund
NHMRC: National Health and Medical Research Council
NIH: National Institutes of Health
NIHR: National Institute of Health Research
REMAP-CAP: Randomised Embedded Multifactorial Adaptive Platform for Community-acquired Pneumonia
ToR: Terms of Reference
UK: United Kingdom
US: United States
